# A multi-gene signature predicts outcome in patients with pancreatic ductal adenocarcinoma

**DOI:** 10.1186/s13073-014-0105-3

**Published:** 2014-12-03

**Authors:** Syed Haider, Jun Wang, Ai Nagano, Ami Desai, Prabhu Arumugam, Laurent Dumartin, Jude Fitzgibbon, Thorsten Hagemann, John F Marshall, Hemant M Kocher, Tatjana Crnogorac-Jurcevic, Aldo Scarpa, Nicholas R Lemoine, Claude Chelala

**Affiliations:** Centre for Molecular Oncology, Barts Cancer Institute, London, United Kingdom; Centre for Tumour Biology, Barts Cancer Institute, London, United Kingdom; Centre for Haemato-Oncology, Barts Cancer Institute, London, United Kingdom; Centre for Cancer and Inflammation, Barts Cancer Institute, London, United Kingdom; ARC-Net Research Centre and Department of Pathology and Diagnostics, University and Hospital Trust of Verona, Verona, Italy

## Abstract

**Background:**

Improved usage of the repertoires of pancreatic ductal adenocarcinoma (PDAC) profiles is crucially needed to guide the development of predictive and prognostic tools that could inform the selection of treatment options.

**Methods:**

Using publicly available mRNA abundance datasets, we performed a large retrospective meta-analysis on 466 PDAC patients to discover prognostic gene signatures. These signatures were trained on two clinical cohorts (*n* = 70), and validated on four independent clinical cohorts (*n* = 246). Further validation of the identified gene signature was performed using quantitative real-time RT-PCR.

**Results:**

We identified 225 candidate prognostic genes. Using these, a 36-gene signature was discovered and validated on fully independent clinical cohorts (hazard ratio (HR) = 2.06, 95% confidence interval (CI) = 1.51 to 2.81, *P* = 3.62 × 10^−6^, *n* = 246). This signature serves as a good alternative prognostic stratification marker compared to tumour grade (HR = 2.05, 95% CI = 1.45 to 2.88, *P* = 3.18 × 10^−5^) and tumour node metastasis (TNM) stage (HR = 1.13, 95% CI = 0.66 to 1.94, *P* = 0.67). Upon multivariate analysis with adjustment for TNM stage and tumour grade, the 36-gene signature remained an independent prognostic predictor of clinical outcome (HR = 2.21, 95% CI = 1.17 to 4.16, *P* = 0.01). Univariate assessment revealed higher expression of *ITGA5*, *SEMA3A*, *KIF4A*, *IL20RB*, *SLC20A1*, *CDC45*, *PXN*, *SSX3* and *TMEM26* was correlated with shorter survival while *B3GNT1*, *NOSTRIN* and *CADPS* down-regulation was associated with poor outcome.

**Conclusions:**

Our 36-gene classifier is able to prognosticate PDAC independent of patient cohort and microarray platforms. Further work on the functional roles, downstream events and interactions of the signature genes is likely to reveal true molecular candidates for PDAC therapeutics.

**Electronic supplementary material:**

The online version of this article (doi:10.1186/s13073-014-0105-3) contains supplementary material, which is available to authorized users.

## Background

Pancreatic ductal adenocarcinoma (PDAC) is amongst the leading causes of cancer deaths in the world, with 5-year survival of less than 5% [1,2]. Surgical excision offers the best chance for long-term survival [3,4] since there is limited response to adjuvant chemotherapy [5,6]. Median survival following surgical resection and adjuvant chemotherapy is between 22 and 24 months [7]. Only 15% of patients present with a resectable tumour. Of these, nearly 80% develop local or distant recurrence within 2 years, reflecting the need for better prognostic and predictive biomarkers to dictate adjuvant therapy [4].

Clinical and pathological characteristics have limited value in predicting prognosis in PDAC patients with metastatic, locally advanced or resectable sub-groups [8,9]. There are no established diagnostic, prognostic or predictive biomarkers for PDAC [10]. Compared to other cancers, such as breast and ovarian cancers, there is no biological or genetic classifier for PDAC tumours, despite the increased understanding of genetic heterogeneity among PDAC tumours [6,11,12]. Recent research has started to discriminate different PDAC subtypes, which indicate patients at a relatively higher risk of metastasis and those with a differential response to therapy [3,6,13–17]. These studies present a complex genomic and transcriptomic landscape for PDAC and they propose gene signatures that are able to predict patient outcome for their respective clinical cohorts. For example, Collisson *et al*. [13] identified a 62-gene expression signature representing three distinct PDAC subtypes, which were assessed for prognostic value in a clinical cohort of only 27 patients. Donahue *et al*. [14] proposed an integrative 171-gene signature (microRNA, DNA copy number and mRNA expression) that was able to stratify PDAC patients into two prognostic subgroups. Of these 171 genes, the independent validation was restricted to three genes only: *SRC* (*n* = 148), *p85α* (*n* = 148) and *CBL* (*n* = 42). Similar efforts by Stratford *et al*. [3] and Zhang *et al*. [17] identified expression prognostic gene signatures, which they validated for 67 and 27 patients, respectively. Biankin *et al*. [6] demonstrated independent prognostic power for four genes in a clinical cohort of 88 patients: *ROBO2*, *ROBO3*, *SEMA3A* and *PLXNA1*. Whilst these studies have produced a series of multi-modal PDAC molecular profiles with potential prognostic ability, cross-validation, generalisability and systematic analysis are lacking, which as yet precludes clinical application.

Since signatures derived through meta-analysis offer increased power and robustness [18–22], we conducted a large-scale retrospective, multi-cohort analysis of PDAC mRNA abundance profiles to identify clinically relevant PDAC prognostic biomarkers. All patients with survival data were included in analyses without screening using any other clinical variable.

## Methods

Additional information about the methods is provided in Additional file 1.

### Literature search

PDAC mRNA abundance datasets were collected through the pancreatic expression database [23]. Studies with both mRNA and clinical data were used for prognostic signature discovery and validation. Studies without clinical data were only used in the cluster analysis of signature genes.

### Verona clinical cohort

Samples from 28 PDAC patients who underwent a surgical resection of PDAC at the University of Verona (Italy) were profiled using Affymetrix GeneChip Human Exon 1.0 ST Array. Data are made available through GEO [24] [GEO:GSE56560]. The samples collected from the Verona cohort were collected in accordance with the Declaration of Helsinki. They were residual tissue samples left over after samples were collected for diagnostic purposes. They were collected with the approval of the Verona Hospital Trust local ethics committee under a general approval to study biomarkers in the pancreas cancer programme 1885. The samples had either individual patient consent or a waiver from the ethics committee (Azienda Ospedaliera Universitaria Integrata Verona, Italy). In both circumstances, the samples were collected and accessed into the biobank. The samples and associated information were anonymised to ensure patient privacy and protection.

### Preprocessing pancreatic ductal adenocarcinoma mRNA abundance datasets

Raw Affymetrix GeneChip Human Exon 1.0 ST, Gene 1.0 ST, U133 Plus 2.0 and U133A Array data were robust multi-array average (RMA) normalised independently. Agilent and Illumina datasets were downloaded in original preprocessed form from GEO [3,6]. Across all datasets, whenever multiple probe sets were mapped to the same HUGO gene nomenclature committee (HGNC) gene symbol, the probe set with the largest variance was kept.

### The Cancer Genome Atlas breast, colorectal and ovarian cancer datasets

Preprocessed The Cancer Genome Atlas (TCGA) breast (BRCA), colorectal (COADREAD) and ovarian (OV) cancer datasets (mRNA abundance and clinical data) were downloaded from TCGA data portal (gdac), release 2014-01-15.

### Differentially expressed features

Differentially expressed genes (transcript cluster ids) between 42 matching PDAC associated normal tissues were identified using LIMMA.

### *In silico* dataset merging

The Verona and Zhang (training) cohorts were merged using the distance weighted discrimination algorithm (DWD).

### Parameter selection

The choice of the optimal parameters (*P*_adjusted_ < 0.01, absolute log_2_-fold change >0, and Wald test *P* < 0.05) was based on the classification performance and signature size for the training cohort (Additional file 2: Table S3).

### Univariate prognostic gene selection

The DWD-merged training cohort was used to estimate the prognostic value of the differentially expressed genes. Patient risk groups were ascertained by median-dichotomising mRNA abundance intensities (continuous) into low- and high-risk groups, and relative hazard was estimated using a Cox proportional hazards model.

### Patient classification

The classification of patients into risk groups was done using prediction analysis of microarrays (PAM). The PAM algorithm clusters samples into *k*-groups using nearest shrunken centroids. Using the DWD-merged mRNA abundance data alongside patient survival data, the model was trained in a leave-one-out cross-validation (LOOCV) setting using R package pamr v1.54.1. The model with the minimum cross-validation error in the training cohort was selected. The trained model was applied to mRNA abundance profiles in the validation cohort to predict patient risk groups, which were subsequently used in Kaplan–Meier analysis. The survival differences between patient risk groups were assessed using a Cox proportional hazards model (hazard ratio), with a *P* value estimated through a Wald test or log-rank test.

### Classification accuracy

The classification accuracy of the validation cohort was estimated by establishing a 2 × 2 *confusion table*. Patients with survival time >20 months (average of median PDAC survival in studies listed in Additional file 2: Table S1 except Donahue *et al*.) were labelled as low-risk group, while the patients with survival time *≤*20 months were classed as high-risk group. Sensitivity, specificity and accuracy were estimated through a 2 × 2 contingency table.

### Quantitative real-time RT-PCR

Quantitative real-time RT-PCR (qRT-PCR) was performed for *ITGA5*, *NOSTRIN*, *CDC45* and *KIF4A* for a mix of 12 samples from the Verona cohort and nine independent new samples.

## Results

### Prognostic assessment of differentially expressed genes in pancreatic ductal adenocarcinoma

To capture PDAC heterogeneity sufficiently well, we conducted a meta-analysis involving 466 PDAC samples from ten mRNA abundance datasets (nine studies) generated on different platforms [3,6,13–17,25,26] (Additional file 2: Table S1). Of these, 316 samples had patient survival data available. To investigate the existence of potential clinical subtypes amongst these PDAC samples, a multi-step supervised feature selection was performed, which identified candidate prognostic genes (Additional file 3: Figure S1 and Additional file 4: Figure S2). Forty-two PDAC samples were initially compared against their matched normal tissues [17]. Having identified 7,374 out of 33,297 differentially expressed transcript clusters (*P*_adjusted_ < 0.01), we sought to establish an association with patient outcome. For increased power and inter-tumour heterogeneity coverage, a merged training cohort (Verona + Zhang cohorts, *n* = 70) was created using the DWD algorithm [27]. The microarray platform similarity between the two cohorts reduced the potential biases arising from *in silico* merging. The training cohort was used to identify statistically significant prognostic genes (Cox proportional hazards model, Wald test *P* < 0.05). Univariate survival analysis revealed 225 highly prognostic genes, which stratified patients into appropriate risk groups (*P* < 0.05) (Additional file 2: Table S2). We comprehensively evaluated the choice of our gene expression and survival analysis cut-offs, and chose the optimal parameters that maximised training cohort performance (Additional file 2: Table S3).

### The 36-gene signature predicts clinical outcome in independent patient studies

**Figure 1:**
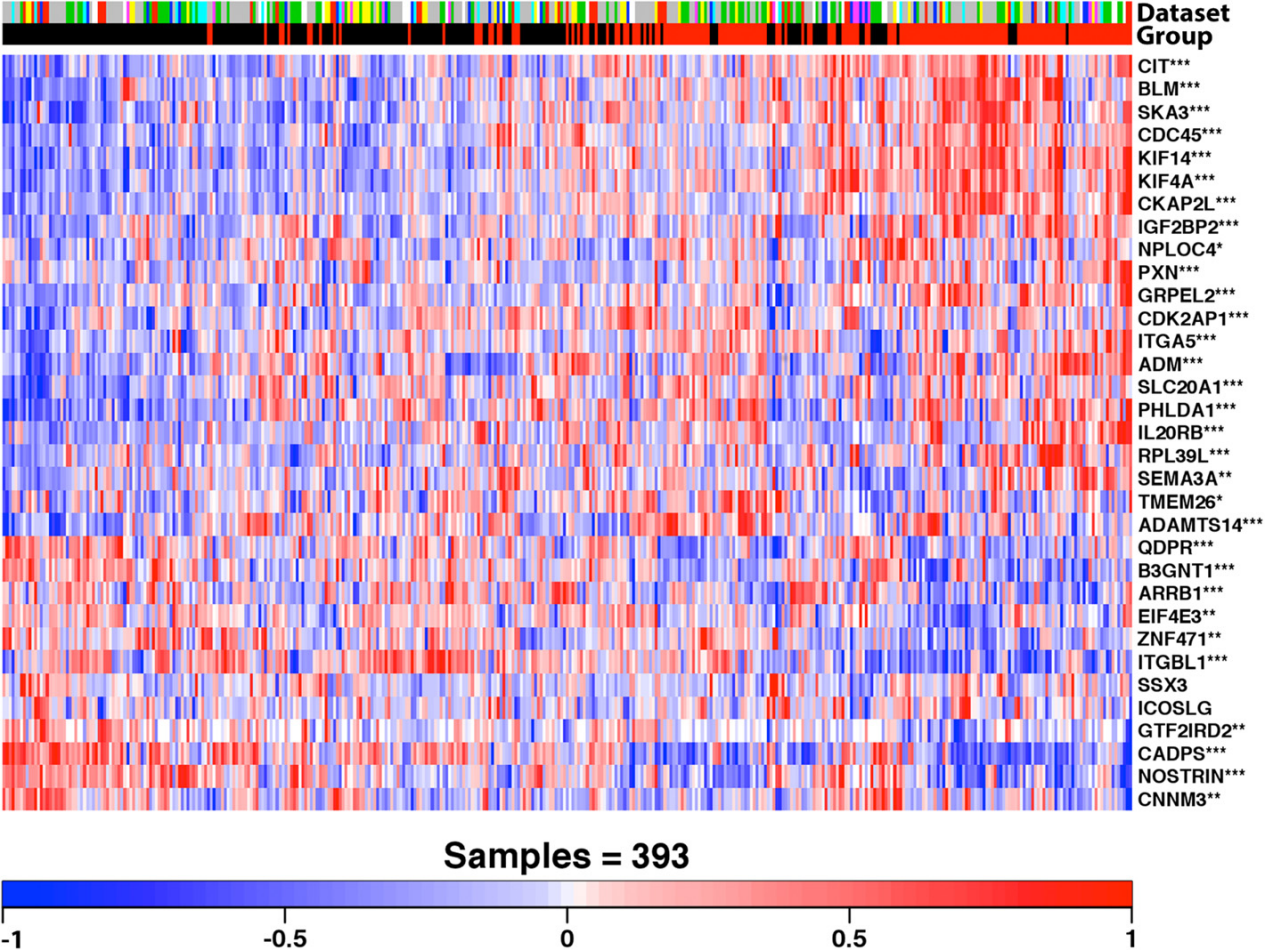
**mRNA abundance patterns of 36-gene signature.** Three genes (*RFX8*, *RPSAP58* and *GTF2IRD2B*) were removed as over 50% of the validation cohort samples did not have corresponding expression profiles available given the annotation libraries used at the time of this study, and were therefore deemed unsuitable for clustering. The annotations on the right represent HGNC gene symbols along with asterisks indicating the significance of the mRNA-based differential expression between the predicted risk groups (***P_adjusted_ < 0.001, **P_adjusted_ < 0.01 and *P_adjusted_ < 0.05). The covariates along the horizontal axis show a patient’s predicted risk group (black is low risk and red is high risk) and underlying dataset (red = Badea, green = Biankin, blue = Collisson, cyan = Donahue, magenta = Grutzmann, yellow = Pei, grey = Stratford and white = Winter). These results show two clusters of differential gene expression between the patient groups that demonstrate significantly different overall survival. HGNC, HUGO Gene Nomenclature Committee.

**Figure 2:**
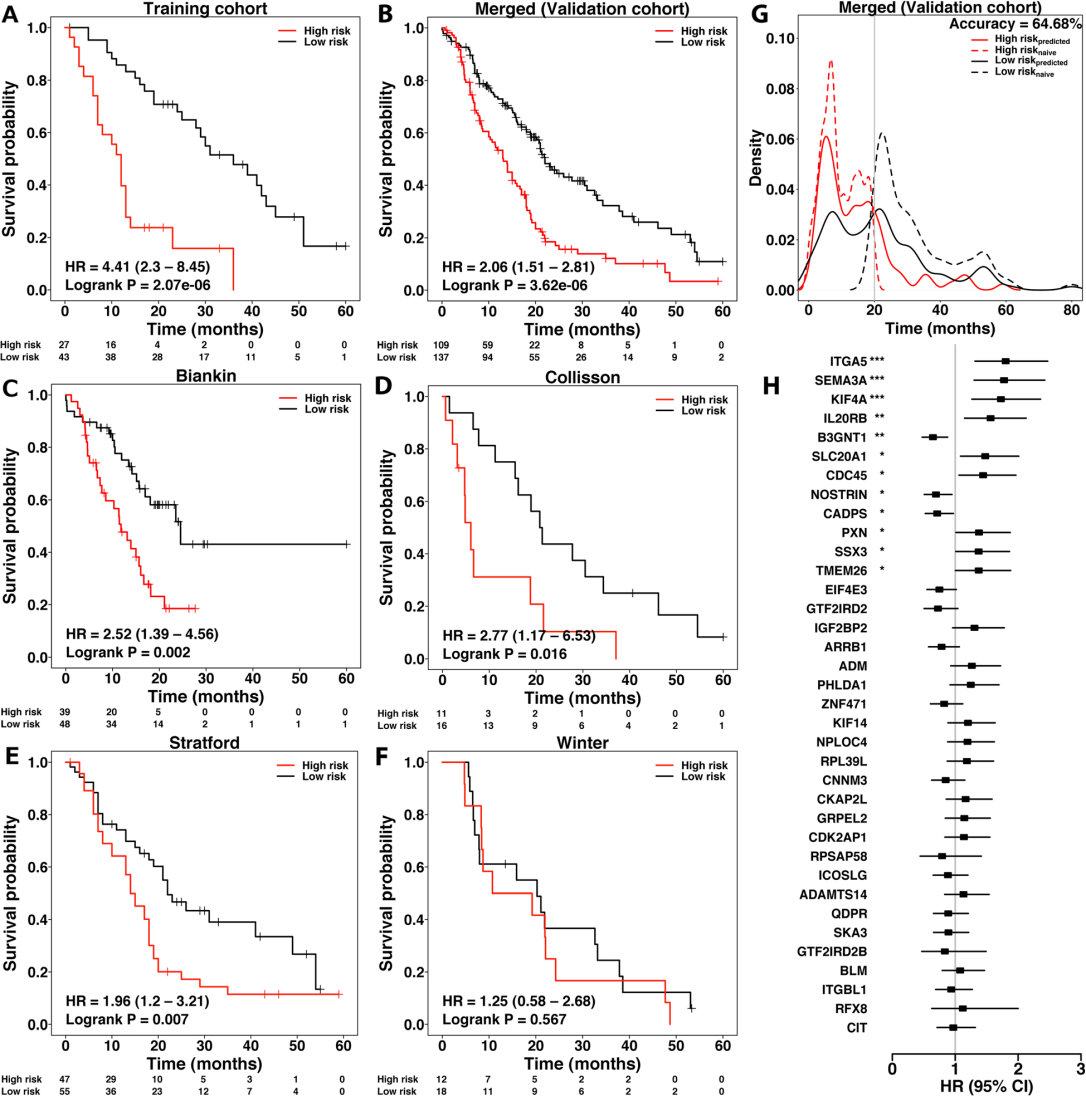
**Performance assessment of PDAC classifier. (A)** Kaplan–Meier survival analysis of patient risk groups identified with the training cohort using the 36-gene signature. **(B)** Kaplan–Meier survival analysis of the predicted risk group of patients in the merged validation cohort using the 36-gene signature. The hazard ratio (HR) was estimated using a Cox proportional hazards model, and curves were compared using a log-rank test. **(C-F)** Independent validation across all the individual datasets that make up the validation cohort. **(G)** Assessment of classification accuracy using sensitivity and specificity estimates. Patients in the validation cohort were dichotomised by a median survival of 20 months (grey line), and classed into low- and high-risk groups, dashed black and red curves respectively. The solid curves represent a patient’s predicted risk group. Comparison between the two sets of groups indicates an overall 64.68% classification accuracy. **(H)** Forest plots of the genes in the prognostic signature. A Cox proportional hazards model was fitted to the signature genes in a univariate context. The horizontal axis represents HR (black squares) and 95% CIs (solid line). The asterisks represent the significance of the difference in patient outcome between the low- and high-expression groups (***P < 0.001, **P < 0.01 and *P < 0.05; Wald test). CI, confidence interval; HR, hazard ratio; PDAC, pancreatic ductal adenocarcinoma.

Using the 225 candidate prognostic genes, we searched for the most discriminating subset of genes that correlated with clinical outcome (overall survival). PAM was used with the mRNA abundance profiles along with the clinical data using the training cohort. By minimising the LOOCV error rate, a 36-gene classifier was built with a prognostic group identification accuracy of 78% (Additional file 5: Figure S3, Additional file 6: Figure S4 and Additional file 2: Tables S4 and S5). Subsequently, this classifier was employed to predict outcome for patients initially assigned to the validation cohorts (Figures 1 and 2A). The 36-gene signature identified patients with a significantly shorter postoperative survival (hazard ratio (HR) = 2.06, 95% confidence interval (CI) = 1.51 to 2.81, *P* = 3.62 × 10^−6^ log-rank test, classification accuracy = 64.68%) (Figure 2B,G). Notably, only up to 17% patients in the high-risk group survived beyond 24 months compared to 45% in the low-risk group. Likewise, the rate of 36-month survival in the high-risk group was only 11% compared to 31% in the low-risk group.

To validate our finding further, Kaplan–Meier analysis was conducted on each of the validation cohort’s constituent datasets independently. The 36-gene signature was significantly associated with patient outcome in the Biankin (HR = 2.52, 95% CI = 1.39 to 4.56, *P* = 0.002, *n* = 87), Collisson (HR = 2.77, 95% CI = 1.17 to 6.53, *P* = 0.016, *n* = 27) and Stratford (HR = 1.96, 95% CI = 1.2 to 3.21, *P* = 0.007, *n* = 102) datasets (Figure 2C,D,E). The combined HR was 2.23, 95% CI was 1.58 to 3.14, *P* = 2.97 × 10^−6^ and accuracy was 66.28%. However, our gene signature was not confirmed in the Winter cohort (HR = 1.25, *P* = 0.567, 95% CI = 0.58 to 2.68, *n* = 30) (Figure 2F). Possible reasons for this discrepancy could be the unbalanced nature of the clinical cohort assembled by Winter *et al*., as none of the patients received adjuvant therapy, and because this was the only cohort in our study with a significantly higher number of Grade 3/4 patients (Additional file 2: Table S1). It is noteworthy that the validation cohort has data from three different microarray platforms (Affymetrix, Agilent and Illumina) (Additional file 2: Table S1). This emphasises the stability and robustness of our signature across the PDAC cohorts used in this analysis.

Since clinical stage for tumour node metastasis (TNM) and tumour grade may also possess prognostic value, we compared the predictive ability of our molecular signature to that of clinical stage and histological grade. Stage IA, IB and IIA patients were compared to stage IIB, III and IV patients, while grade 1 and 2 patients were compared to grade 3 and 4 patients. Kaplan–Meier survival analysis of these datasets revealed TNM stage as a poor prognostic factor (HR = 1.13, 95% CI = 0.66 to 1.94, *P* = 0.667) (Additional file 7: Figure S5), whereas tumour grade was a strong predictor of patient outcome (HR = 2.05, 95% CI = 1.45 to 2.88, *P* = 3.18 × 10^−5^) (Additional file 8: Figure S6). These findings are in line with previously published pancreatic cancer studies suggesting the strong prognostic value of tumour grade and highly variable patient survival within TNM stage groups [28,29]. Following the univariate analysis of stage and grade, the 36-gene signature classifier was adjusted for the effect of stage, grade and combined effect of both stage and grade. The multivariate modelling results further supported the contention that the 36-gene signature is an independent predictor of patient outcome (stage-adjusted model: HR = 1.94, 95% CI = 1.32 to 2.85, *P* = 7.9 × 10^−4^; grade-adjusted model: HR = 2.03, 95% CI = 1.33 to 3.09, *P* = 9.77 × 10^−4^; stage-and-grade-adjusted model: HR = 2.21, 95% CI = 1.17 to 4.16, *P* = 0.014; Wald test *P* values). The prognostic capability of the 36-gene signature was further compared to a panel of 15 clinicopathological covariates [6]. Our signature outperformed all 15 covariates, including the resection margins, and was the best prognostic indicator (*P*_Margins_ = 0.0094, *n* = 131; *P*_36-sig_ = 0.002, *n* = 87).

### Subtype-specific patterns of gene expression and outcome association

To understand the PDAC subtype-specific transcriptional activity, we asked whether these 36 genes are differentially expressed between the two PDAC subtypes. To avoid training-specific bias, we limited our analysis to the validation datasets only (*n* = 393). Of the 36 genes, 31 were differentially expressed (*P*_adjusted_ < 0.05) (Figure 1, Additional file 2: Table S6). However, the reproducibility of the training-set-derived centroids in the validation cohort suggests their stability across a number of patients studies conducted on different array platforms. To assess prognostic power of the signature genes, we conducted a univariate survival analysis restricted to the validation cohort (*n* = 246). The results indicated there were 12 significantly prognostic genes (*P* < 0.05; Wald test) (Figure 2H, Additional file 2: Table S7). Of these, the higher expression of *ITGA5*, *SEMA3A*, KIF4A, *IL20RB*, *SLC20A1*, *CDC45*, *PXN*, *SSX3* and *TMEM26* was correlated with poor survival, suggesting oncogenic potential (Figures 1 and 2H). Conversely, *B3GNT1*, *NOSTRIN* and *CADPS* followed a reverse trend with down-regulation associated with poor outcome (Figures 1 and 2H), thus suggesting a tumour suppressor role.

### Comparison with pancreatic ductal adenocarcinoma prognostic gene signatures

**Figure 3:**
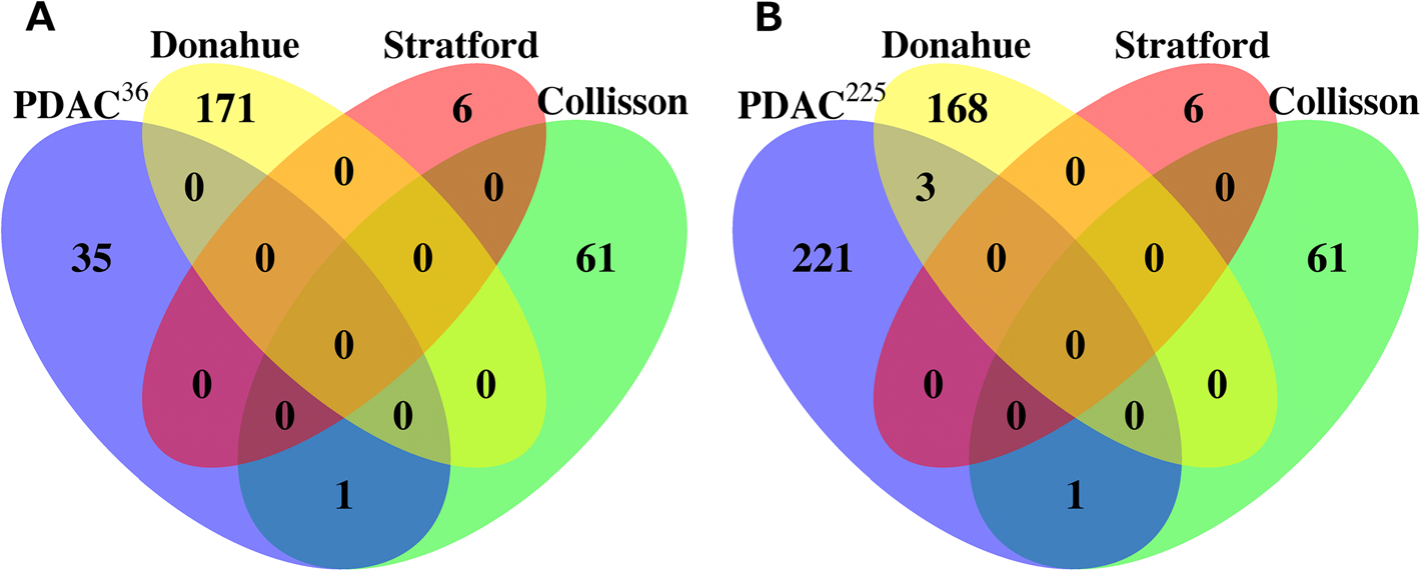
**Overlap among PDAC gene signatures. (A)** Venn diagram detailing overlaps between the 36-gene signature and existing PDAC gene signatures. **(B)** Same as **(A)** except all the candidate prognostic genes used to derive the 36-gene signature were assessed for overlap with the existing PDAC gene signatures. PDAC, pancreatic ductal adenocarcinoma.

A number of PDAC prognostic gene signatures have been proposed and most of the underlying datasets were included in our analyses [3,13,14,16]. With regards to existing classifiers, the performance of the 36-gene signature was comparable to the 62-gene PDAssigner [13] (*P* = 0.038, *n* = 27), 171-composite gene signature [14] (*P* = 0.009, *n* = 25) and six-gene signature [3] (*P* = 0.001, *n* = 67). In terms of underlying genes, the overlap between the 36-gene signature and known PDAC gene sets was non-existent, and so was the trend amongst these gene sets (Figure 3A) [3,6,13–17,25,26]. There was only one gene (*PHLDA1*) in common between the 36-gene signature and the *Quasimesenchymal* subtype (QM-PDA) of Collisson *et al*. Upon expanding the 36-gene signature to its candidate prognostic gene list (225 genes, hereafter referred to as PDAC-225) (Additional file 2: Table S2), only three genes were shared between PDAC-225 and Donahue *et al.* (Figure 3B). Extending the analysis to single gene predictors (*DPEP1* and *TPX2* for Zhang *et al*., and *ROBO2*, *ROBO3*, *PLXNA1* and *SEMA3A* for Biankin *et al.*), again, there was no overlap between either of these gene sets and the rest of the previously published results. Apart from *SEMA3A*, none of the Zhang *et al.* and Biankin *et al.* genes were in common with the 36-gene and PDAC-225 signatures.

### Random gene signatures of pancreatic ductal adenocarcinoma

**Figure 4:**
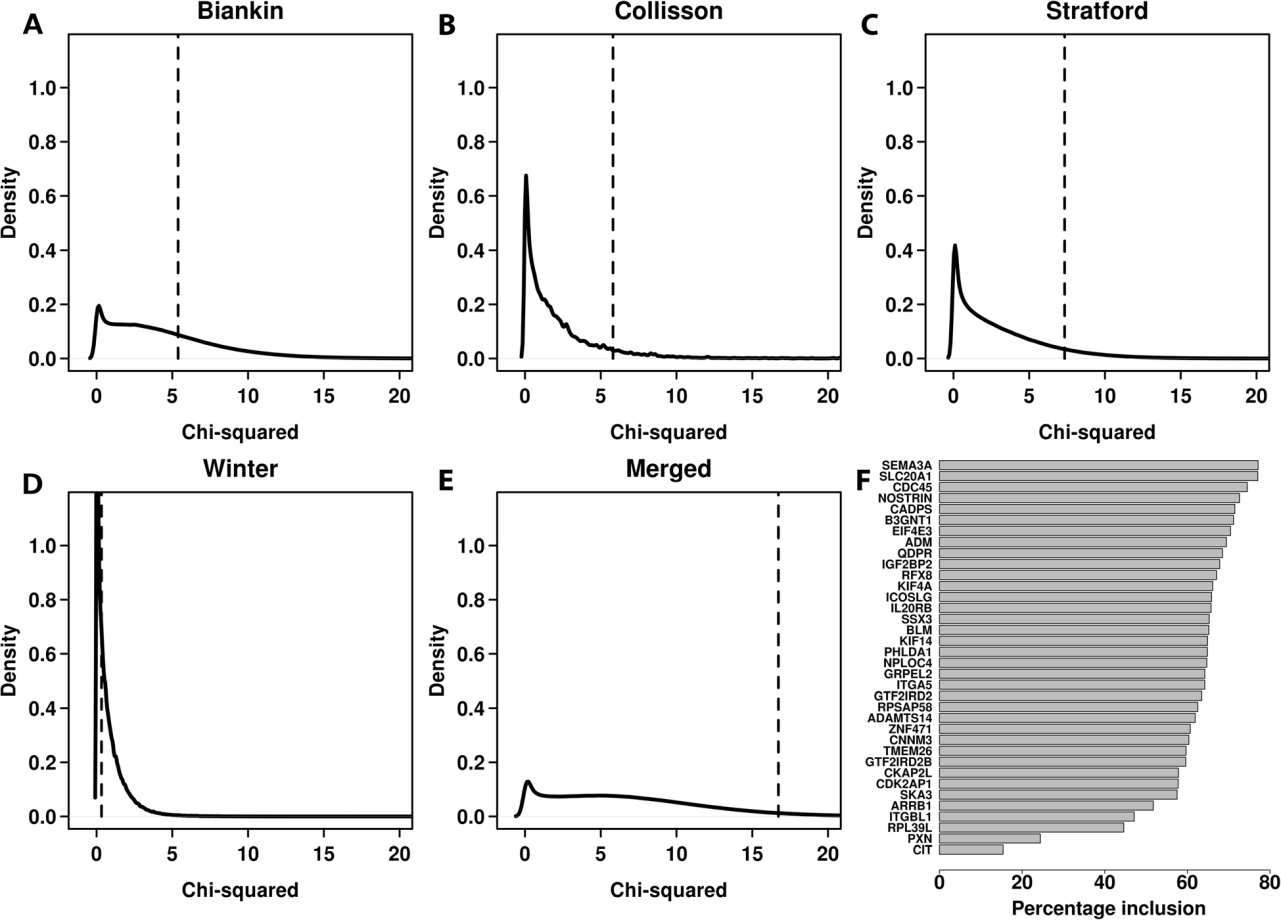
**Random prognostic gene signatures for PDAC. (A–E)** Randomisation results of 5 million gene signatures with the initial feature set of 225 genes. All signatures were trained and validated on the same datasets as used for the derivation of the 36-gene signature. A χ^2^ statistic was used a performance metric for comparing random signatures with the 36-gene signature. The dashed lines are the χ^2^ values for the 36-gene signature. Overall, only 0.19% of the random signatures outperformed the 36-gene signature across all datasets. **(F)** Percentage inclusion of genes in the 36-gene signature for the randomly selected gene signatures. Thirty-one out of 36 genes were amongst the top 5% genes with highest inclusion frequency, with *SEMA3A* ranked highest (overall ranked 11th). PDAC, pancreatic ductal adenocarcinoma.

Previous studies have exposed a large number of verifiable random gene signatures in breast and non-small-cell lung cancer, which explains the lack of overlap between prognostic gene signatures [20,30]. However, it is unknown whether PDAC expression datasets contain any valid random prognostic signatures. To determine the presence of such signatures, and further investigate these for potential enrichment of the 36 prognostic genes, we generated over 5 million random gene signatures. Since the 36-gene signature was created using an initial pool of 225 prognostic genes, we randomly selected 225 genes and processed these using the same protocol as used for the identification of the 36-gene signature. The performance of these signatures was assessed with the validation datasets alone as well as with the merged validation cohort using a χ^2^ statistic. In total, 1,138 signatures were significantly associated with patient outcome in each of the Biankin, Collisson and Stratford cohorts (*P*_adjusted_ < 0.05) (Additional file 9: Figure S7). None of the signatures were reproducible in the Winter cohort following adjustment for multiple comparisons. As shown in the kernel density plots (Figure 4) for individual validation set studies, the 36-gene signature demonstrated superior performance compared to most random gene signatures. In conclusion, the 36-gene signature represents an optimal combination of highly reproducible and robust prognostic genes.

### Functional interpretation and validation

Functional analysis of the 225 candidate genes revealed a number of highly enriched mitosis and cell division checkpoint sub-networks (*P*_adjusted_ = 8.88 × 10^−3^) (Additional file 2: Table S8). Focussing on the biological pathways represented by the 36-gene signature, we found 13 significantly enriched pathways (*P* = 0.004 to 0.047), primarily encompassing cell cycle, cell-cell signalling and cell survival and death processes (Additional file 2: Table S9). Since cell cycle is central to tumour development and progression, we tested the prognostic value of the 36-gene signature in three other disease types: breast, colorectal and ovarian cancers [31–33]. In breast cancer, 11 genes were associated with overall survival (*P* < 0.05) (Additional file 2: Table S10). However, both colorectal and ovarian cancers presented an entirely different clinical association of these genes with only one gene in each cancer type (colorectal: *B3GNT1*, ovarian: *PXN*) associated with poor prognosis (Additional file 2: Tables S11 and S12).

We validated the expression of four prognostic genes (*ITGA5*, *KIF4A*, *CDC45* and *NOSTRIN*) using qRT-PCR in a mix of profiled and independent samples. The results confirmed the prognostic trend from the array data. Higher expression of *ITGA5*, *KIF4A* and *CDC45* was correlated with poor prognosis while low expression of *NOSTRIN* was correlated with poor prognosis (Additional file 10: Figure S8).

## Discussion

Our systematic analysis yields a clinically valuable and biologically plausible 36-gene signature, which predicts outcome after current surgical treatment irrespective of the expression platform used. This prognostic gene-expression signature was derived from two patient cohorts, and validated on four fully independent patient cohorts treated in North America, Europe and Australia, and profiled using different microarray platforms, thus demonstrating its robustness across clinical spectrum and assay platforms. Our meta-analysis approach offered greater statistical power in addressing PDAC biological heterogeneity as well as treatment variation. The treatment variation and sample size may influence the prognostic subtype identification process, as shown recently in a breast cancer study of 2,000 samples indicating ten subgroups, which were potentially suitable for different approaches to treatment [34]. However, given that there are only limited treatment options for PDAC and our limited understanding of the predisposing risk factors at the molecular level [35], low- and high-risk grouping is, perhaps, most tractable from a clinical point of view.

We also demonstrate a lack of overlap between existing PDAC gene signatures that show prognostic and/or predictive potential [3,6,13–17,25,26]. This could be due to the small discovery cohort size, the inherent noise in different microarray experiments leading to confounded results [36], and/or, the impact of the clinicopathological characteristics of samples selected for a particular study on candidate gene selection. We addressed these issues with systematic preprocessing of disparate microarray datasets and subsequent integration of results while keeping noise aggregation to a minimum. The integration of clinical cohorts with different treatment regimens or histological subtypes remains an influential factor when isolating disease-specific genes. Most patients included in this meta-analysis had localised PDAC, with a similar spread of clinical covariates, and were treated with surgical resection along with adjuvant chemotherapy, thus, ensuring that our integration of disparate patient cohorts was adequately addressed. Taking these factors together with the results of the permutation analysis showing the existence of 1,138 signatures, the lack of overlap between existing PDAC signatures can be explained as previously shown for breast and lung cancers [20,30].

The relatively sparse clinical annotation made comparison with currently used clinicopathological predictors difficult. TNM stage and tumour grade, the most commonly used clinical predictors, were available for a limited number of patients and our 36-gene signature predictor was superior to stage-based and highly competitive with grade-based classifiers. Further validation of our marker in an adequately powered study is needed.

Upon assessing the prognostic ability of the 36 genes independently, and examining the pathways they modulate, we demonstrated significant enrichment of *ITGA5* and *SEMA3A* in a number of signalling pathways, including the recurrently mutated axonal guidance-signalling pathway [6]. The correlation of increased *SEMA3A* expression with poor survival of patients with PDAC is supported by two previous studies [6,37], possibly due to the ability of *SEMA3A* to promote PDAC cell invasion [37]. Our independent validation of *ITGA5*, *KIF4A*, *CDC45* and *NOSTRIN* was based on the biological plausibility and their relatively greater contribution to this predictive gene signature. Our top hit, *ITGA5*, encodes for integrin alpha-5, which in turn associates with integrin beta-1 to form the fibronectin receptor (*α5β1*), and higher expression levels correlate with metastatic potential and poor prognosis in patients with PDAC [38]. Kinesin family member 4A (*KIF4A*), a microtubule-binding motor protein, is a candidate oncogene identified in lung cancer [39]. *CDC45* plays a critical role in DNA replication; thus, its expression is associated with rapidly proliferating cell populations [40]. Cdc45 is also a critical effector of *Myc*-dependent DNA replication stress and thus, when over-expressed or amplified, could act as an oncogene [41]. *CDC45* plays a critical role in DNA replication and its expression is tightly associated with proliferating cell populations [6,37]. Lastly, we confirmed that *NOSTRIN* expression could be a good prognosticator. NOSTRIN is an F-BAR-domain-containing protein, a group of adaptor proteins performing essential roles, such as membrane protrusion and migration, in conjunction with FGFR1, Rac1 and Sos1 [42]. The control of FGFR1 sub-cellular location is vital for invasion and metastases in PDAC [43].

Thus far, none of the identified mutations in PDAC, exemplified by *K-RAS*, the most frequent mutation, have borne therapeutic targets [44]. The altered expression of the genes identified herein, in the absence of mutations, may be more useful for identifying drug targets [45–47] and will need to be explored in experimental studies. Moreover, the presence of *ADM*, *B3GNT1*, *CNNM3*, *ICOSLG*, *ITGA5*, *KIF4A* and *QDPR* in the urine and/or plasma proteome, lead us to believe that in our gene signature there are potentially interesting clinically valuable prognostic biomarkers [48,49]. Finally, our classifier’s ability to predict accurately across different array platforms confirms its robustness, irrespective of clinical presentation or medical delivery systems. Thus, we anticipate a thorough examination of functional roles of these genes and corresponding downstream events to reveal novel PDAC diagnostic and therapeutics.

## Conclusions

Here we present a large retrospective meta-analysis of 466 PDAC patients to discover prognostic gene signatures with 5-year overall survival as an end point. These signatures were trained on two patient cohorts (*n* = 70), validated on four patient cohorts (*n* = 246), and examined for cross-platform reproducibility. We observe that the higher expression of *ITGA5*, *SEMA3A*, *KIF4A*, *IL20RB*, *SLC20A1*, *CDC45*, *PXN*, *SSX3* and *TMEM26* was correlated with shorter survival while down-regulation of *B3GNT1*, *NOSTRIN* and *CADPS* was associated with poor outcome. Our 36-gene classifier is able to prognosticate PDAC independent of patient cohort and microarray platforms. Further work on the functional roles, downstream events and interactions of the signature genes is likely to reveal true molecular candidates for PDAC therapeutics.

## Additional files

Additional file 1:
**Supplementary Methods.**
Additional file 2: Table S1. PDAC studies, along with corresponding platform and cohort size. Rows in grey indicate the discovery and training datasets. **Table S2.** Results for the 36-gene signature for the univariate Cox proportional hazards model. Only genes that are significantly associated with patient outcome (survival time) are listed (*P* < 0.05; Wald test). The columns contain hazard ratio (HR), 95% confidence intervals (HR95L: lower and HR95U: upper), Wald test *P* values (*P*) and total number of samples in the training cohort (*n*). **Table S3.** Class-wise error rate determined by varying feature selection parameters. Parameters tested were differential expression variables (LIMMA *P*_adjusted_ and absolute log_2_-fold change) and Wald test *P* following a univariate Cox proportional hazards fit (training cohort only). The model with the smallest error rate and gene-set size was selected, and subsequently applied to independent validation cohorts. **Table S4.** HGNC genes (Gene) and gene descriptions (Description) selected by the prognostic classifier. **Table S5.** Centroids for the low- and high-risk groups estimated by the nearest shrunken centroid fit on the training cohort. **Table S6.** Differential mRNA abundance analysis of 36-gene signature for the validation cohort. The columns indicate LIMMA statistics including *P*_adjusted_ and log_2_-fold change. The last column (ID.with.stars) shows the *P*_adjusted_ derived significance (*** *P*_adjusted_ < 0.001, ** *P*_adjusted_ < 0.01 and * *P*_adjusted_ < 0.05). **Table S7.** Results for the 36-gene signature for the univariate Cox proportional hazards model with the validation cohort. The columns contain hazard ratio (HR), 95% confidence intervals (HR95L and HR95U), Wald test *P* values (*P*) and total number of samples in the validation cohort (*n*). Thirty-two out of 36 genes were present in all validation datasets. **Table S8.** Enrichment analysis of gene subsets found in 225 candidate prognostic genes. Genes were analysed using GeneMania. No additional neighbouring genes were added to the network. The columns contain functionally related gene sets (Feature), the enrichment significance of genes found in 225 candidate genes containing known functionally related genes (FDR), number of related genes found in 225 candidate genes (Genes in network), and the overall total number of genes associated with a particular function in the human genome. **Table S9.** Pathway enrichment analysis of 36 genes using the Ingenuity IPA tool. Pathways are ranked by the significance of enrichment (−log_10_(*P*)). **Table S10.** Results for the 36-gene signature for the univariate Cox proportional hazards model with the TCGA breast cancer cohort (BRCA). The columns contain hazard ratio (HR), 95% confidence intervals (HR95L and HR95U), Wald test *P* values (*P*) and total number of samples in the cohort (*n*). **Table S11.** Results for the 36-gene signature for the univariate Cox proportional hazards model with the TCGA colorectal cancer cohort (COADREAD). The columns contain hazard ratio (HR), 95% confidence intervals (HR95L and HR95U), Wald test *P* values (*P*) and total number of samples in the cohort (*n*). **Table S12.** Results for the 36-gene signature for the univariate Cox proportional hazards model results with the TCGA ovarian cancer cohort (OV). The columns contain hazard ratio (HR), 95% confidence intervals (HR95L and HR95U), Wald test *P* values (*P*) and total number of samples in the cohort (*n*). **Table S13.** Primers used for qRT-PCR. Additional file 3: Figure S1. Signature identification process. From the Zhang dataset, 7,374 differentially expressed transcript clusters (TCs) were identified (*P*_adjusted_ < 0.01) and 225 significantly prognostic genes were identified by fitting a univariate Cox proportional hazards model to the merged Verona and Zhang cohorts (training datasets). A 36-multi-gene classifier was trained using the training datasets, and subsequently applied to the validation datasets to predict patient risk score. Risk scores were assessed for their prognostic power using Kaplan–Meier survival analysis. The survival curves were compared using a log-rank test. DE, differentially expressed; LOOCV, leave-one-out cross-validation; PAM, prediction analysis of microarrays; PDAC, pancreatic ductal adenocarcinoma; TC(I), transcript cluster (Identifier). Additional file 4: Figure S2. PRISMA flow chart showing study selection steps for this meta-analysis. PDAC, pancreatic ductal adenocarcinoma. Additional file 5: Figure S3. Overall and class-wise error as a function of classifier size (number of genes). The horizontal axis (both top and bottom panels) represents the threshold (delta) values limiting the number of genes in the nearest shrunken centroid fit. The vertical axis (both top and bottom panels) shows the cross-validation classification error by varying the delta. Asterisks show the optimal performance in the top panel. In the bottom panel, lines 1 (red) and 2 (green) show class-wise predictive performance (training cohort) for the high- and low-risk groups, respectively. Additional file 6: Figure S4. Heat map of mRNA abundance intensities of 36-gene signature applied to the training cohort. RMA preprocessed and DWD merged data (Verona and Zhang cohorts) were transformed to *z* scores (data shown as rows in the heatmap). The legend represents relative over- (red) and under-expression (blue). The covariates at the top represent predicted low- (black) and high-risk (red) patients. DWD, distance weighted discrimination algorithm; RMA, robust multi-array average. Additional file 7: Figure S5. Kaplan–Meier survival analysis to assess prognostic value of TNM stage. **(A-D)** Patients were assigned to low- (stage IA/IB/IIA) and high-risk (IIB/III/IV) groups, and a Cox proportional hazards model was fitted. None of the datasets showed a significant difference in patient survival. **(E)** Patient outcome between all stage groups was compared using a log-rank test. Stage-specific groups did not have significantly different prognosis (*P* = 0.87, log-rank test). HR, hazard ratio; TNM, tumour node metastasis. Additional file 8: Figure S6. Kaplan–Meier survival analysis to assess prognostic value of tumour grade. **(A–E)** Grade 1 and 2 patients were compared to grade 3 and 4 patients using a Cox proportional hazards model. Tumour grade had a significant association with patient survival (*P* = 3.18 × 10^−5^, log-rank test). The prognostic value of grade was modestly reproducible across all individual clinical cohorts. **(F)** Patient outcome was compared between multiple grade groups. Although largely dominated by grade 2 and 3 patients, the difference in patient outcome for these groups was highly significant (*P* = 4.31 × 10^−4^, log-rank test). HR, hazard ratio. Additional file 9: Figure S7. Comparison of random gene signatures significantly associated with patient prognosis for each validation cohort (*P*_adjusted_ < 0.05). None of the signatures were reproducible in the Winter cohort following adjustment of the *P* values for multiple comparisons. Additional file 10: Figure S8. RT-PCR results for genes *ITGA5*, *KIF4A*, *CDC45* and *NOSTRIN*. HR, hazard ratio; OS, overall survival.

## Abbreviations

CI: confidence interval
DWD: distance weighted discrimination algorithm
HR: hazard ratio
HGNC HUGO: Gene Nomenclature Committee
OS: overall survival
PAM: prediction analysis of microarrays
PDAC: pancreatic ductal adenocarcinoma
qRT-PCR: quantitative real-time reverse-transcription polymerase chain reaction
RMA: robust multi-array average
TC: transcript cluster
TCGA: The Cancer Genome Atlas
TNM: tumour node metastasis
