# Supplementary material for: A multi-gene signature predicts outcome in patients with pancreatic ductal adenocarcinoma
Source: Genome Med. 2014 Dec 3;6(12):105. doi: 10.1186/s13073-014-0105-3 (PMC4293116; doi:10.1186/s13073-014-0105-3)
Supplement: Additional file 1: — Supplementary Methods. [file 13073_2014_105_MOESM1_ESM.docx]

# A multi-gene signature predicts outcome in patients with pancreatic ductal adenocarcinoma

Syed Haider^1,†^, Jun Wang^1^, Ai Nagano^1^, Ami Desai^2^, Prabhu Arumugam^2^, Laurent Dumartin^1^, Jude Fitzgibbon^3^, Thorsten Hagemann^4^, John F Marshall^2^, Hemant M Kocher^2^, Tatjana Crnogorac-Jurcevic^1^, Aldo Scarpa^5^, Nicholas R Lemoine^1,†^, Claude Chelala^1,†^

^1^ Centre for Molecular Oncology, Barts Cancer Institute, London, United Kingdom

^2^ Centre for Tumour Biology, Barts Cancer Institute, London, United Kingdom

^3^ Centre for Haemato-Oncology, Barts Cancer Institute, London, United Kingdom

^4^ Centre for Cancer and Inflammation, Barts Cancer Institute, London, United Kingdom

^5^ARC-Net Research Centre and Department of Pathology and Diagnostics, University and Hospital Trust of Verona, Verona, Italy

† Corresponding authors

### Additional file 1: Methods

### Preprocessing PDAC mRNA abundance datasets

Raw data of the Verona cohort were preprocessed using the Affymetrix power tools (APT) package (v1.14.3). The samples were RMA-normalized and both the exon-level as well as gene-level intensities were restricted to core annotation level using the Affymetrix exon array computational tool (ExACT) library (v2). Filters for undetected probesets (detected above background; P < 0.05 for at least 50% samples) and cross-hybridization were applied to ensure that only reliable probesets are kept and subsequently quantified into the corresponding gene’s expression[[1](#_ENREF_1)]. Similarly, gene-level intensities were further restricted by applying probeset-level (DABG P < 0.05 for at least 50% of the constituting probesets) and sample level (expressed in at least 50% of the samples in each group) filtering. The transcript cluster id to HGNC gene symbol mappings was done using NetAffy annotations (HuEx-1_0-st-v2 Hg19).

Raw Affymetrix GeneChip Human Genome 1.0 ST, U133 Plus 2.0 and U133A Array data were RMA normalized independently in R statistical environment (v2.14.1) (R packages: oligo v1.18.1, affy v1.32.1, hgu133plus2.db v2.6.3, hgu133a.db v2.6.3, pd.hugene.1.0.st.v1 v3.4.0 and hugene10sttranscriptcluster.db v8.0.1)[[2-7](#_ENREF_2)]. Agilent and Illumina datasets were downloaded in original preprocessed form from Gene Expression Omnibus website[[8](#_ENREF_8), [9](#_ENREF_9)]. The probeset to HGNC gene symbol mappings were done using the respective annotation database (R packages: hgug4112a.db v2.6.3 and illuminaHumanv4.db v1.12.1). Across all datasets, wherever multiple probesets were mapped to the same HGNC gene symbol, the probeset with the largest variance was kept.

### Differentially expressed features

Differentially expressed genes (Transcript cluster ids) between 42 matching PDAC associated normal tissues were identified using LIMMA in R statistical environment (v.2.14.1) (R package: limma v3.10.3).

### In-silico dataset merging

The Verona cohort (Affy HuEx 1.0 ST) and Zhang cohort (Affy HuGene 1.0 ST) were merged using Distance Weighted Discrimination algorithm (DWD) in R statistical environment (v.2.14.1) (R packages: inSilicoMerging v0.3.0 and DWD v0.10).

### Parameter selection

Of the various combinations of LIMMA P_adjusted_ (< 0.05, 0.01), LIMMA expression absolute log_2_-fold change (> 0, 0.585, 1) and significance of prognostic ability using Wald test P (< 0.0.5, 0.01, 0.001), the choice of the most optimal parameters (P_adjusted_ < 0.01, absolute log_2_-fold change > 0, and Wald test P < 0.05) was based on the classification performance and signature size on the training cohort (Supplementary Table 3).

### Univariate prognostic gene selection

The merged Verona and Zhang cohort (n = 70) was used to estimate the prognostic value of all the differentially expressed genes. Patient risk groups were ascertained by median-dichotomising mRNA abundance intensities into low- and high-risk groups, and relative hazard was estimated. The Cox proportional hazards model was fitted to every gene independently in R statistical environment (v2.14.1) (R package: survival v2.36-14).

### Patient classification

The patient risk group classification was done using a model trained on the training cohort (n = 70) with Prediction Analysis of Microarrays (PAM). The PAM algorithm clusters the samples into two k-groups using nearest shrunken centroids. The model was trained in a leave-one-out-cross-validation (LOOCV) setting. The trained model was applied to mRNA abundance profiles in the validation cohort to predict patient risk groups, which were subsequently used in Kaplan-Meier analysis. These analyses including survival modeling and Kaplan-Meier analysis were conducted in R statistical environment (v.2.14.1) (R packages: pamr v1.54 and survival v2.36-14). All Kaplan-Meier P values reported in the manuscript were estimated using Logrank test, unless stated/annotated otherwise. The survival data for Biankin et al was downloaded from the ICGC DCC portal (dcc.icgc.org) (v8).

### Classification accuracy

Classification accuracy of the validation cohort was estimated by establishing 2x2 *confusion table* of true positives, false positives, true negatives and false negatives. Patients with survival time > 20 months (average of median PDAC survival in studies listed in Supplementary Table 1 except Donahue *et al*) were labeled as a low-risk group, while the patients with survival time *≤* 20 months were classed as a high-risk group. Patients with censored data below the PDAC median survival were removed from this analysis due to lack of clarity on the risk class assignment. Sensitivity, Specificity and Accuracy was estimated through 2x2 contingency table of true positives, false positives, true negatives and false negatives.

### Functional analysis and annotation data

The functional analyses of the 36 genes were conducted using IPA (Ingenuity® Systems, [www.ingenuity.com](http://www.ingenuity.com)) and Pancreatic Expression Database[[10-12](#_ENREF_10)].

### Randomisation

We generated over five million random genesets of size 225 (without replacement) from the same pool of 13,568 genes that was used for the development of the 36-gene classifier. These genesets were used to derive five million unique prognostic gene classifiers trained on Verona and Zhang cohorts, and subsequently validated on Biankin, Collisson, Stratford and Winter cohorts. For a random gene *Gx,* the probability to be selected by a random geneset would be P(*Gx*) = 225/13568 = 0.017. Given P(*Gx*), gene *Gx* would be sampled by a total of *Gx_Total_ =*85,000 genesets. The inclusion percentage for a gene was estimated as a fraction of *Gx_Total_* based on how many significant prognostic signatures (P_adjusted_ < 0.05 in the merged validation cohort) contained that gene. Logrank P values were adjusted for multiple comparisons using the Benjamini and Hochberg method.

### Visualizations

All visualizations including heatmaps, density plots, Kaplan-Meier plots and forest plots were produced in R statistical environment (v.2.14.1) (R packages: gplots v2.10.1, Heatplus v2.1.0 and rmeta v2.16).

### Quantitative real-time RT-PCR (qRT-PCR)

To validate the expression trend of the top prognostic markers, qRT-PCR was performed in a mix of 12 samples from the Verona cohort and nine independent new samples. Briefly, total RNA was isolated from frozen tissues via phenol extraction (TRIzol Reagent; Invitrogen Corp., Carlsbad, CA). Total RNA was reversely transcribed using QuantiTect Reverse Transcription kit (Qiagen). The expression of selected genes *CDC45*, *NOSTRIN*, *ITGA5* and *KIF4A* and housekeeping control genes S16, *RPLP0* and *HPRT1* were detected on a 7500 Real-Time PCR system (Applied Biosystems) using SYBR Green dye (Qiagen) (Primers provided in Supplementary Table 13).

### Supplementary Figures and Tables

### Supplementary Figure 1:

Schematic view of the signature identification process. 7,374 differentially expressed transcript clusters (TC) were identified on the Zhang dataset (P_adjusted_<0.01). 225 significantly prognostic genes were identified by fitting a univariate Cox proportional hazards model to the merged Verona and Zhang cohorts (Training datasets). A 36 multi-gene classifier was trained using the training datasets, and subsequently applied to the validation datasets to predict patient risk score. Risk scores were assessed for their prognostic power using Kaplan-Meier survival analysis. The survival curves were compared using Logrank test.

### Supplementary Figure 2:

PRISMA[[13](#_ENREF_13)] flow chart showing study selection steps for this meta-analysis.

### Supplementary Figure 3:

Overall and class-wise error as a function of classifier size (number of genes). The horizontal axis (both top and bottom panel) represents the threshold (delta) values limiting the number of genes in the nearest shrunken centroid fit. The vertical axis (both top and bottom panel) shows the cross validation classification error by varying the delta. Asterisks show the most optimal performance in the top panel. In bottom panel, 1 (red) and 2 (green) shows class-wise predictive performance (training cohort) high- and low-risk groups respectively.

### Supplementary Figure 4:

Heatmap of mRNA abundance intensities of 36-gene signature applied to the training cohort. RMA preprocessed and DWD merged data (Verona and Zhang cohorts) was transformed to z-scores (data shown as rows in the heatmap). The legend represents relative over- (red) and under-expression (blue). The covariates at the top represent predicted low- (black) and high-risk (red) patients.

### Supplementary Figure 5:

Kaplan-Meier survival analysis to assess prognostic value of TNM stage. **(A-D)** Patients were assigned to low- (Stage IA/IB/IIA) and high-risk (IIB/III/IV) groups, and Cox proportional hazards model was fit. None of the datasets showed significant difference in patient survival. **(E)** Patient outcome between all stage groups was compared using Logrank test. Stage-specific groups did not have significantly different prognosis (P = 0.87, Logrank test).

### Supplementary Figure 6:

Kaplan-Meier survival analysis to assess prognostic value of tumour grade. **(A-E)** Grade 1 and 2 patients were compared to grade 3 and 4 using Cox proportional hazards model. Tumour grade significant association with patient survival (P = 3.18 x 10^-5^, Logrank test). The prognostic value of grade was modestly reproducible across all individual clinical cohorts. **(F)** Patient outcome between multiple grade groups was compared. Although largely dominated by grade 2 and 3 patients, the difference in patient outcome for these groups was highly significant (P = 4.31 x 10^-4^, Logrank test).

### Supplementary Figure 7:

Comparison of random gene signatures that were significantly associated with the patient prognosis in each of the validation cohorts (P_adjusted_ < 0.05). None of the signatures were reproducible in the Winter cohort following adjustment of the P values for multiple comparisons.

### Supplementary Figure 8:

RT-PCR results for genes: *ITGA5*, *KIF4A, CDC45* and *NOSTRIN*.

### Supplementary Table 1:

List of PDAC studies, along with corresponding platform and cohort size. Rows in grey indicate the discovery/training datasets.

### Supplementary Table 2:

Univariate Cox proportional hazards model results of 36-gene signature. Only genes that are significantly associated with patient outcome (survival time) are listed (P < 0.05; Wald test). The columns contain hazard ratio (HR), 95% confidence intervals (HR95L and HR95U), Wald test P values (P) and total number of samples in the training cohort (n).

### Supplementary Table 3:

Class-wise error rate by varying feature selection parameters. Parameters tested were differential expression variables (Limma P_adjusted_ and absolute log_2_-fold change) and Wald test P following univariate Cox proportional hazards fit (training cohort only). The model with the smallest error rate and geneset size was selected, and subsequently applied to independent validation cohorts.

### Supplementary Table 4:

List of HGNC gene names (Gene), and gene description (Description) selected by the prognostic classifier.

### Supplementary Table 5:

Centroids of low- and high-risk groups estimated by the nearest shrunken centroids fit on the training cohort.

### Supplementary Table 6:

Differential mRNA abundance analysis of 36-gene signature on the validation cohort. The columns indicate LIMMA statistics including P_adjusted_ and log_2_ fold change. The last column (ID.with.stars) is P_adjusted_ derived significance keys (*** = P_adjusted_ < 0.001, ** = P_adjusted_ < 0.01 and * = P_adjusted_ < 0.05).

### Supplementary Table 7:

Univariate Cox proportional hazards model results of 36-gene signature in the validation cohort. The columns contain hazard ratio (HR), 95% confidence intervals (HR95L and HR95U), Wald test P values (P) and total number of samples in the validation cohort (n). Thirty-two out of 36 genes were present in all validation datasets.

### Supplementary Table 8:

Enrichment analysis of gene subsets found in 225 candidate prognostic genes. Genes were analysed using GeneMania [[14](#_ENREF_14)]. No additional neighbouring genes were added to the network. The columns contain functionally related genesets (Feature), enrichment significance of genes found in 225 candidate genes containing known functionally related genes (FDR), number of related genes found in 225 candidate genes (Genes in network), and the overall total number of genes associated with a particular function in the human genome.

### Supplementary Table 9:

Pathway enrichment analysis of 36 genes using Ingenuity IPA tool. Pathways are ranked by the significance of enrichment (-log_10_(P)).

### Supplementary Table 10:

Univariate Cox proportional hazards model results of 36-gene signature in TCGA breast cancer cohort (BRCA). The columns contain hazard ratio (HR), 95% confidence intervals (HR95L and HR95U), Wald test P values (P) and total number of samples in the cohort (n).

### Supplementary Table 11:

Univariate Cox proportional hazards model results of 36-gene signature in TCGA colorectal cancer cohort (COADREAD). The columns contain hazard ratio (HR), 95% confidence intervals (HR95L and HR95U), Wald test P values (P) and total number of samples in the cohort (n).

### Supplementary Table 12:

Univariate Cox proportional hazards model results of 36-gene signature in TCGA ovarian cancer cohort (OV). The columns contain hazard ratio (HR), 95% confidence intervals (HR95L and HR95U), Wald test P values (P) and total number of samples in the cohort (n).

### Supplementary Table 13:

Primers used for qRT-PCR.

### References

1. Lockstone HE: **Exon array data analysis using Affymetrix power tools and R statistical software.** *Brief Bioinform* 2011, **12:**634-644.

2. Collisson EA, Sadanandam A, Olson P, Gibb WJ, Truitt M, Gu S, Cooc J, Weinkle J, Kim GE, Jakkula L, et al: **Subtypes of pancreatic ductal adenocarcinoma and their differing responses to therapy.** *Nature medicine* 2011, **17:**500-503.

3. Badea L, Herlea V, Dima SO, Dumitrascu T, Popescu I: **Combined gene expression analysis of whole-tissue and microdissected pancreatic ductal adenocarcinoma identifies genes specifically overexpressed in tumor epithelia.** *Hepatogastroenterology* 2008, **55:**2016-2027.

4. Donahue TR, Tran LM, Hill R, Li Y, Kovochich A, Hargan Calvopina J, Patel SG, Wu N, Hindoyan A, Farrell JJ, et al: **Integrative Survival-Based Molecular Profiling of Human Pancreatic Cancer.** *Clinical cancer research : an official journal of the American Association for Cancer Research* 2012.

5. Grutzmann R, Pilarsky C, Ammerpohl O, Luttges J, Bohme A, Sipos B, Foerder M, Alldinger I, Jahnke B, Schackert HK, et al: **Gene expression profiling of microdissected pancreatic ductal carcinomas using high-density DNA microarrays.** *Neoplasia* 2004, **6:**611-622.

6. Pei H, Li L, Fridley BL, Jenkins GD, Kalari KR, Lingle W, Petersen G, Lou Z, Wang L: **FKBP51 affects cancer cell response to chemotherapy by negatively regulating Akt.** *Cancer Cell* 2009, **16:**259-266.

7. Zhang G, Schetter A, He P, Funamizu N, Gaedcke J, Ghadimi BM, Ried T, Hassan R, Yfantis HG, Lee DH, et al: **DPEP1 inhibits tumor cell invasiveness, enhances chemosensitivity and predicts clinical outcome in pancreatic ductal adenocarcinoma.** *PLoS One* 2012, **7:**e31507.

8. Stratford JK, Bentrem DJ, Anderson JM, Fan C, Volmar KA, Marron JS, Routh ED, Caskey LS, Samuel JC, Der CJ, et al: **A six-gene signature predicts survival of patients with localized pancreatic ductal adenocarcinoma.** *PLoS Med* 2010, **7:**e1000307.

9. Biankin AV, Waddell N, Kassahn KS, Gingras MC, Muthuswamy LB, Johns AL, Miller DK, Wilson PJ, Patch AM, Wu J, et al: **Pancreatic cancer genomes reveal aberrations in axon guidance pathway genes.** *Nature* 2012.

10. Chelala C, Hahn SA, Whiteman HJ, Barry S, Hariharan D, Radon TP, Lemoine NR, Crnogorac-Jurcevic T: **Pancreatic Expression database: a generic model for the organization, integration and mining of complex cancer datasets.** *BMC Genomics* 2007, **8:**439.

11. Dayem Ullah AZ, Cutts RJ, Ghetia M, Gadaleta E, Hahn SA, Crnogorac-Jurcevic T, Lemoine NR, Chelala C: **The pancreatic expression database: recent extensions and updates.** *Nucleic acids research* 2014, **42:**D944-949.

12. Cutts RJ, Gadaleta E, Hahn SA, Crnogorac-Jurcevic T, Lemoine NR, Chelala C: **The Pancreatic Expression database: 2011 update.** *Nucleic acids research* 2011, **39:**D1023-1028.

13. Liberati A, Altman DG, Tetzlaff J, Mulrow C, Gotzsche PC, Ioannidis JP, Clarke M, Devereaux PJ, Kleijnen J, Moher D: **The PRISMA statement for reporting systematic reviews and meta-analyses of studies that evaluate health care interventions: explanation and elaboration.** *PLoS Med* 2009, **6:**e1000100.

14. Warde-Farley D, Donaldson SL, Comes O, Zuberi K, Badrawi R, Chao P, Franz M, Grouios C, Kazi F, Lopes CT, et al: **The GeneMANIA prediction server: biological network integration for gene prioritization and predicting gene function.** *Nucleic acids research* 2010, **38:**W214-220.
