# Supplementary figures and images for: A multi-gene signature predicts outcome in patients with pancreatic ductal adenocarcinoma
Source: Genome Med. 2014 Dec 3;6(12):105. doi: 10.1186/s13073-014-0105-3 (PMC4293116; doi:10.1186/s13073-014-0105-3)

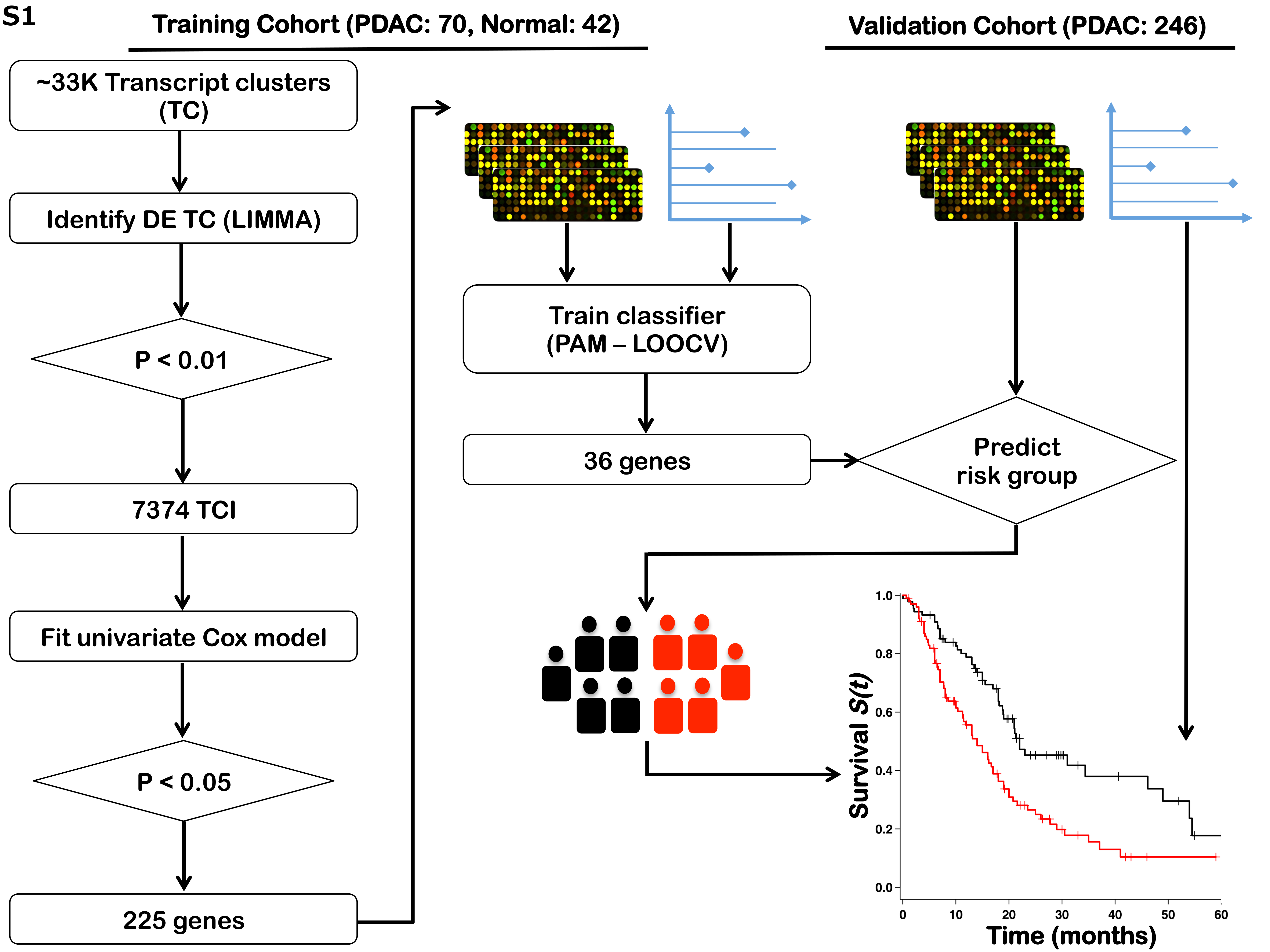

Supplement: Additional file 3: Figure S1. — Signature identification process. From the Zhang dataset, 7,374 differentially expressed transcript clusters (TCs) were identified (P adjusted < 0.01) and 225 significantly prognostic genes were identified by fitting a univariate Cox proportional hazards model to the merged Verona and Zhang cohorts (training datasets). A 36-multi-gene classifier was trained using the training datasets, and subsequently applied to the validation datasets to predict patient risk score. Risk scores were assessed for their prognostic power using Kaplan–Meier survival analysis. The survival curves were compared using a log-rank test. DE, differentially expressed; LOOCV, leave-one-out cross-validation; PAM, prediction analysis of microarrays; PDAC, pancreatic ductal adenocarcinoma; TC(I), transcript cluster (Identifier). [file 13073_2014_105_MOESM3_ESM.tiff]

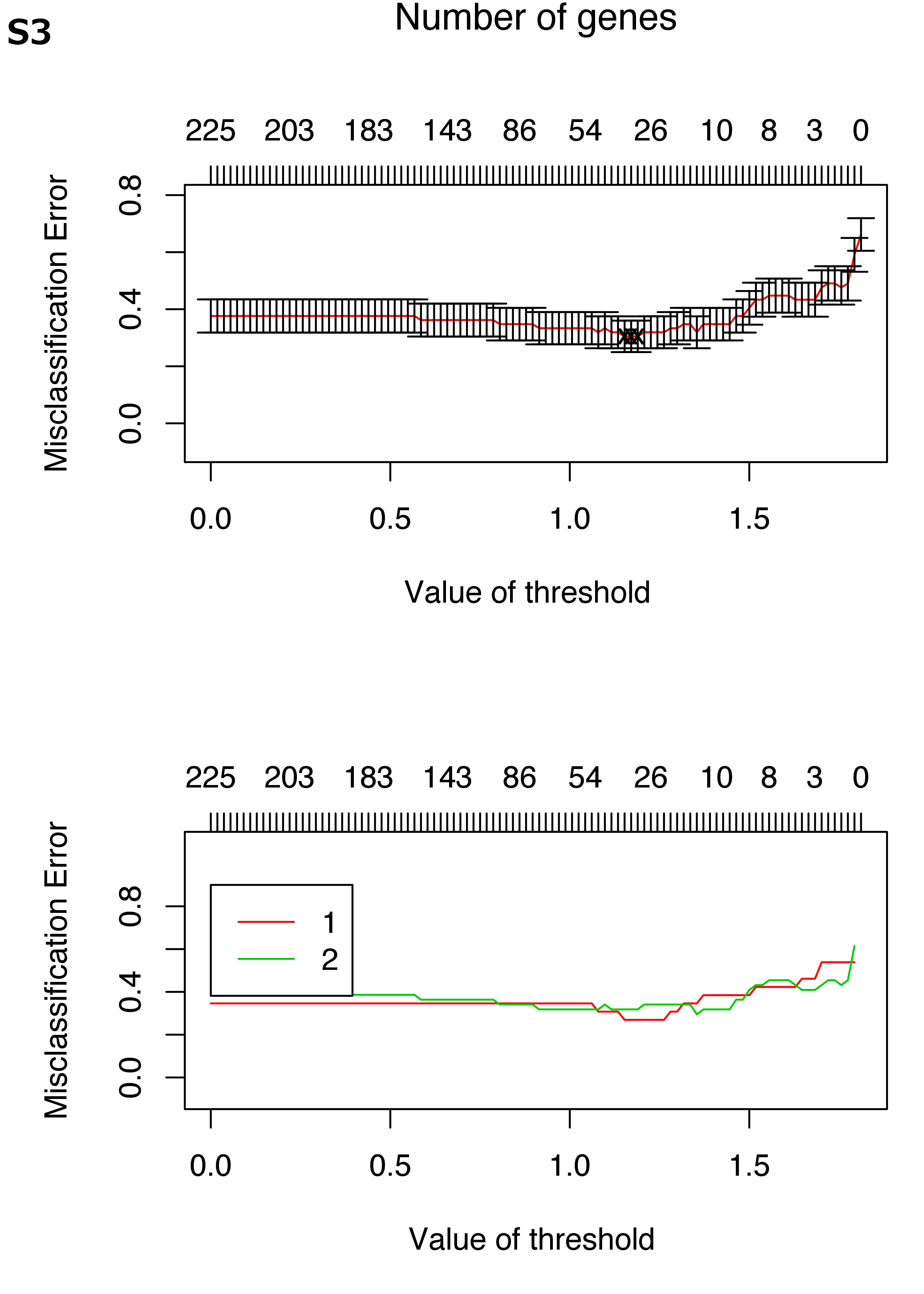

Supplement: Additional file 5: Figure S3. — Overall and class-wise error as a function of classifier size (number of genes). The horizontal axis (both top and bottom panels) represents the threshold (delta) values limiting the number of genes in the nearest shrunken centroid fit. The vertical axis (both top and bottom panels) shows the cross-validation classification error by varying the delta. Asterisks show the optimal performance in the top panel. In the bottom panel, lines 1 (red) and 2 (green) show class-wise predictive performance (training cohort) for the high- and low-risk groups, respectively. [file 13073_2014_105_MOESM5_ESM.tiff]

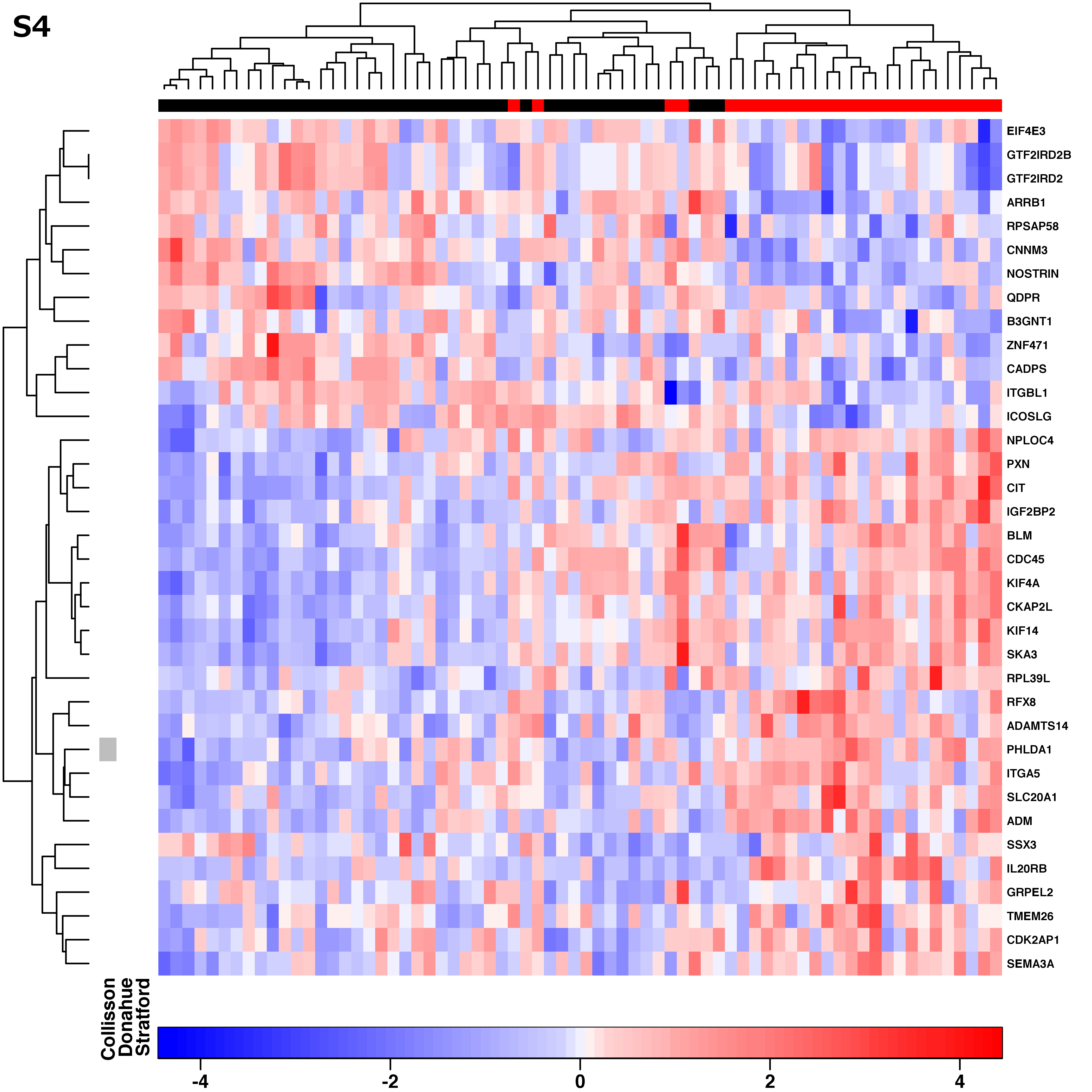

Supplement: Additional file 6: Figure S4. — Heat map of mRNA abundance intensities of 36-gene signature applied to the training cohort. RMA preprocessed and DWD merged data (Verona and Zhang cohorts) were transformed to z scores (data shown as rows in the heatmap). The legend represents relative over- (red) and under-expression (blue). The covariates at the top represent predicted low- (black) and high-risk (red) patients. DWD, distance weighted discrimination algorithm; RMA, robust multi-array average. [file 13073_2014_105_MOESM6_ESM.tiff]

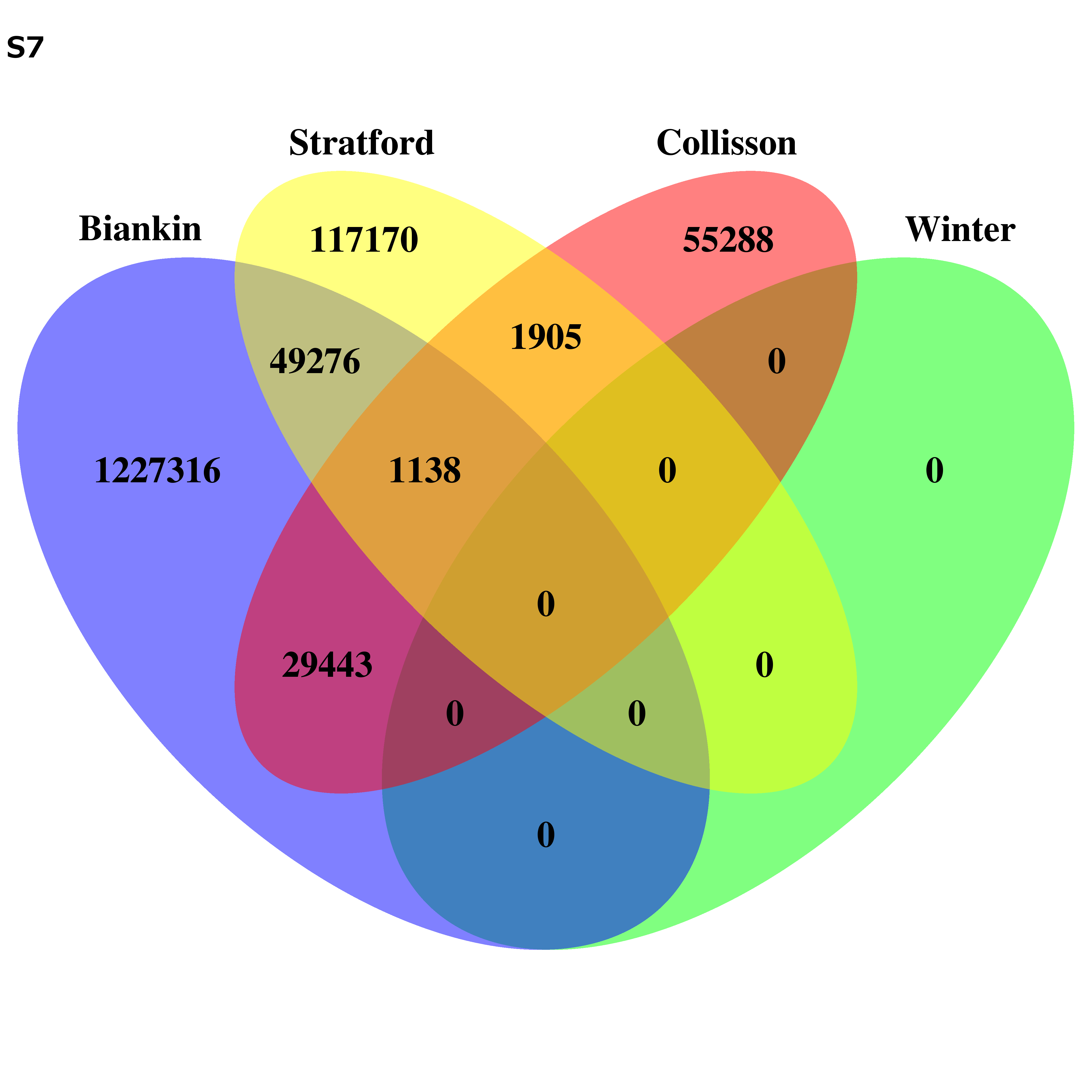

Supplement: Additional file 9: Figure S7. — Comparison of random gene signatures significantly associated with patient prognosis for each validation cohort (P adjusted < 0.05). None of the signatures were reproducible in the Winter cohort following adjustment of the P values for multiple comparisons. [file 13073_2014_105_MOESM9_ESM.tiff]

**S8****ITGA5**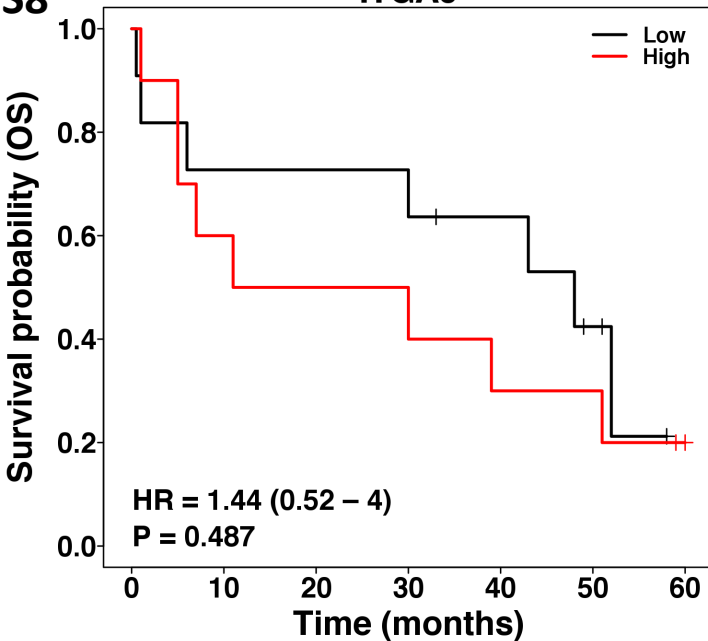**KIF4A**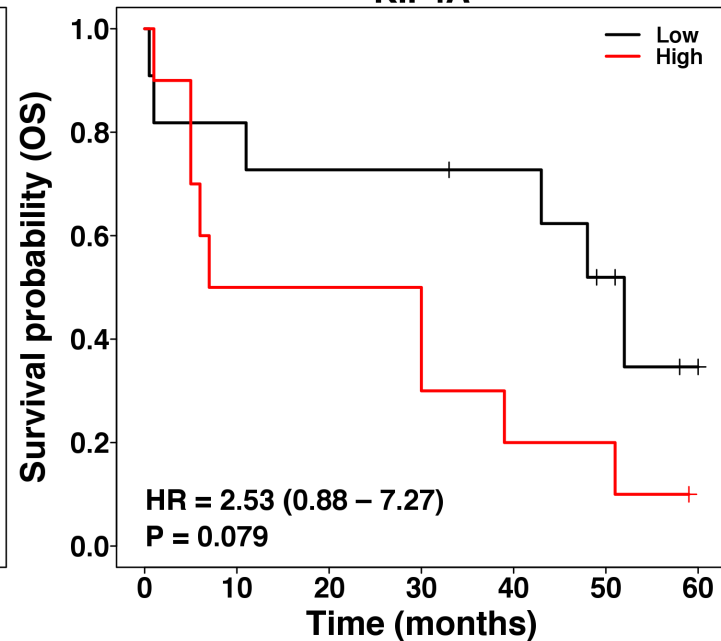**CDC45**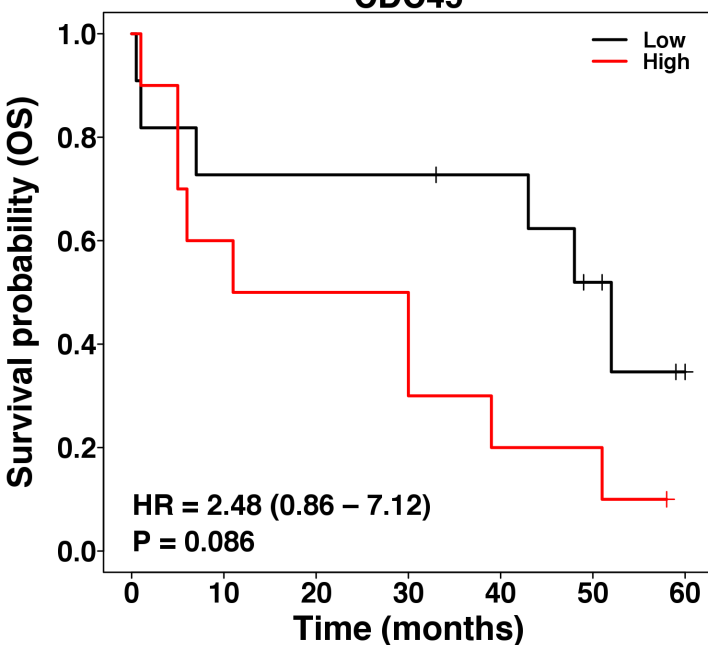**NOSTRIN**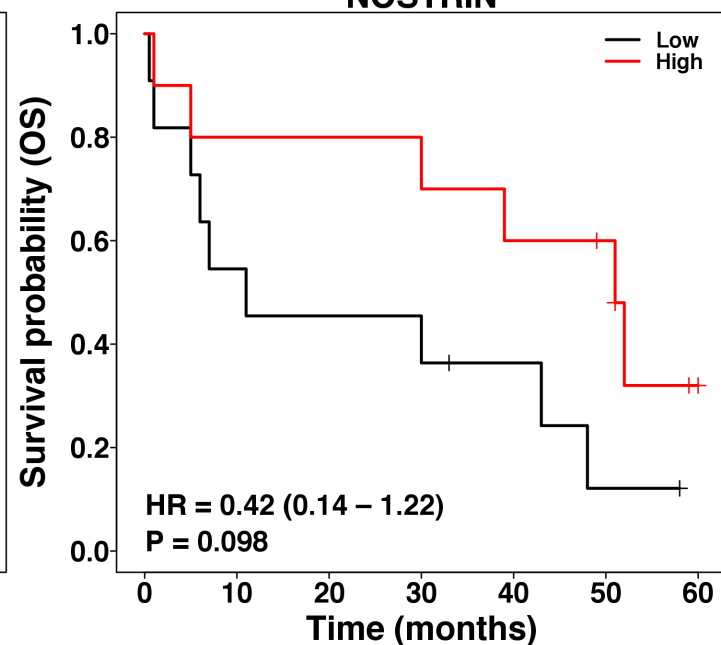

Supplement: Additional file 10: Figure S8. — RT-PCR results for genes ITGA5, KIF4A, CDC45 and NOSTRIN. HR, hazard ratio; OS, overall survival. [file 13073_2014_105_MOESM10_ESM.pdf]
